# Regionalizing land use impacts on farmland birds

**DOI:** 10.1007/s10661-015-4448-z

**Published:** 2015-05-09

**Authors:** Michael Glemnitz, Peter Zander, Ulrich Stachow

**Affiliations:** Leibniz Centre for Agricultural Landscape Research (ZALF), Eberswalder Str. 84, 15374 Müncheberg, Germany

**Keywords:** Impact assessment, Biodiversity, Scenario analysis, Land use pattern, Habitat suitability

## Abstract

The environmental impacts of land use vary regionally. Differences in geomorphology, climate, landscape structure, and biotope inventories are regarded as the main causes of this variation. We present a methodological approach for identifying regional responses in land use type to large-scale changes and the implications for the provision of habitat for farmland birds. The methodological innovations of this approach are (i) the coupling of impact assessments with economic models, (ii) the linking of cropping techniques at the plot scale with the regional distribution of land use, and (iii) the integration of statistical or monitoring data on recent states. This approach allows for the regional differentiation of farmers’ responses to changing external conditions and for matching the ecological impacts of land use changes with regional environmental sensitivities. An exemplary scenario analysis was applied for a case study of an area in Germany, assessing the impacts of increased irrigation and the promotion of energy cropping on farmland birds, evaluated as a core indicator for farmland biodiversity. The potential effects on farmland birds were analyzed based on the intrinsic habitat values of the crops and cropping techniques. The results revealed that the strongest decrease in habitat availability for farmland birds occurred in regions with medium-to-low agricultural yields. As a result of the limited cropping alternatives, the increase in maize production was highest in marginal regions for both examined scenarios. Maize production replaced many crops with good-to-medium habitat suitability for birds. The declines in habitat quality were strongest in regions that are not in focus for conservation efforts for farmland birds.

## Introduction

The decline in biological diversity in European agricultural landscapes is well documented in regional and Europe-wide studies (Defra 2013). Projections of future development predict further declines (Pereira et al. 2010b) to be strongest in areas cropped with annual crops (de Baan et al. 2013). This trend has triggered intense discussions concerning the evaluation of agricultural development options and the use of political and economic tools to steer land use management in a more biodiversity-friendly direction. The recent reform of Europe’s Common Agricultural Policy (CAP), for example, includes the so-called “greening” regulations, which aim to foster biodiversity in Europe’s agricultural areas.

At the national level, biodiversity strategies and action plans (National Biodiversity Strategies and Action Plans (NBSAPS)) have been developed in 180 countries across the world (CBD 2014). Most of these NBSAPS also address agricultural lands. The national biodiversity strategy for Germany (BMU 2007) uses farmland birds as an indicator for the overall biodiversity on agricultural lands. The strategy aims at a return in populations of farmland birds to approximately 90 % of their levels in 1975. However, the mid-term evaluation of the German National Biodiversity Strategy (BMU 2010) has revealed that recent land use changes have caused a further deterioration in the status of farmland birds as opposed to contributing to an improvement. This decreasing trend has not been reversed or halted by major investments in agri-environmental schemes across Europe over the previous two decades (Whittingham 2011).

Birds on farmlands have exhibited the largest declines compared to birds that prefer other habitats (Defra 2013). Shifts in agricultural management are a plausible explanation for the observed declines in farmland bird populations (Chamberlain et al. 2000). The decline has been caused by several factors: changes in arable and grassland management, intensification of farming, and removals of non-cropped habitats (Butler et al. 2010; Defra 2013; Langgemach and Ryslavy 2010). According to Anderson and Fergusson (2006), it will be impossible to understand or predict the implications of land use changes without considering (i) the effects of the crops themselves in terms of the crop vegetation structure and crop management (“intrinsic value” of the crops), together with (ii) the habitat value of the replaced land use type (indirect land use change) and with (iii) the landscape-scale effects. From studies on single bird species, it is well documented that the habitat selection of bird species varies among crop species and is highly related to the vegetation structure of the crops and their dynamics (Eraud and Boutin 2002; Donald 2004; Hoffmann et al. 2013). Additionally, the composition and abundance of weed vegetation within a crop influence habitat selection (Geiger et al. 2014). For the majority of bird species, a minimum vegetation structure is desired for predator avoidance (Lima and Dill 1990). Vegetation height and density are also limiting factors to food search times (Butler et al. 2005; Whittingham et al. 2006). Changes between crop species (e.g., from summer to winter cereals) have a serious impact on food availability (Geiger et al. 2014). Pesticide use affects bird communities more indirectly via the density of invertebrates available for feeding chicks and thus influences breeding success (Hallmann et al. 2014; Brickle and Harper 2002; Rands 1986). Birds search for food over a wide area and are able, at least temporarily, to exploit alternative food resources in cases of disturbance or land use change (Chamberlain et al. 2000). Negative effects of land management practices may become exacerbated when land use changes are associated with the loss of non-cropped habitats (Chamberlain et al. 2000; Gevers et al. 2011). There is a growing concern that the environmental effects of land use on farmland birds will be modified by the surrounding landscape (Danhardt et al. 2010; Gevers et al. 2011; Chamberlain et al. 2000).

Meaningful strategies to halt biodiversity losses must be based on cause–effect analyses and knowledge of the interdependencies of driving forces. Recent agricultural developments in Europe, such as energy cropping, are connected to increased input efficiency and thus support higher levels of specialized production systems. The term “intensification” is thus used to describe the impact of several interconnected mechanisms (Chamberlain et al. 2000), which may, in fact, individually have different effects on species. These driving forces encompass several spatial and temporal scales, from individual farming activities, e.g., soil tillage (Cunningham et al. 2004) and insecticide application (Brickle and Harper 2002), to changes in crop rotations (Smith et al. 2010) and the spatial distributions of crops and farming systems within agricultural landscapes (Danhardt et al. 2010; Chamberlain et al. 2000). Declines in bird species may be significantly correlated to these environmental factors; if agricultural systems are changing, several of these factors are changing simultaneously and are thus correlated with each other. This correlation has important consequences for determining the appropriate scientific approach to studying cause–effect relationships and also for identifying meaningful scales at which countermeasures to halt species decline are most effective. For example, even though soil tillage and insecticide applications harm bird populations, these factors often are proximate causes that are embedded within crop production systems and, in turn, may reflect economic constraints upon the farmers, which themselves serve as the ultimate causes.

The difference between the scale of environmental impacts and the detail of knowledge concerning ecological interactions (Legendre and Legendre 1998) calls for integrated assessment and modeling approaches, which include scales relevant to the impacts on birds, as mentioned above, as well as the integration of economic farm decision-making with the possible consequences for the farming system, crop selection, and sequences of farming activities employed on the fields, which can all affect the habitat quality for bird species. In particular, the role of the spatial arrangement of land use and the underlying socioeconomic impacts on wildlife status are hardly known (Leenhardt et al. 2011). Only a very few agro-environmental studies have accounted for both the coherence and the spatial variability of cropping techniques (Leenhardt et al. 2011). The majority of the spatial land use models consider either evenly or randomly distributed land uses or use synthetic land use data (Bergez et al. 2010). The coupling of human–environment subsystems and the assessment of their spatially explicit outcomes are the major challenge for maintaining the ecosystem services that society values from environmental subsystems (Turner et al. 2007).

Land use change is simply regarded as a change between land cover types (forest, grassland arable, set aside, settlements) in the majority of studies (Leadley et al. 2010; Pereira et al. 2010a), disregarding the changes in crop production techniques within the arable land. In this paper, we argue that the management practice employed on arable fields is a key variable influencing the biodiversity of agricultural landscapes and must be analyzed in further detail in relation to the habitat requirements of target species (e.g., birds) as well as with regard to the regional land use options. For adjustments of conservation measures, it is crucial to know if the bird abundance on a farmed land is limited by the lack of food, the density of the crop vegetation, a mechanical disturbance, or any other variable that can be modified by management. Farm management follows a combination of agronomic and economic principles concerned with the trade-offs between the revenues from the harvest and the costs of the inputs. Consequently, the habitat suitability of a crop field for a species can be seen as the result of a set of economic and agronomic constraints that act in combination to guide distinct farm management systems. Hence, while the direct causes of habitat unsuitability may be identified as agricultural practices, the drivers of these management practices are related to economic constraints. We combine these constraints in a farm model that generates site-specific crop rotations and crop production systems. Information concerning the specific crop and the management system allows for the evaluation of the development of the specific crop field as a potential bird habitat.

In this paper, we present a methodological approach that integrates the agricultural expertise on farmers’ economic behaviors, spatial land use distributions, and spatially explicit impact assessments. For important farmland bird species, we model the match between the specific habitat requirements of the bird species and the habitat suitability as provided by various crop species. We focus on a large-scale assessment of a farmland bird indicator for arable land for the German federal state of Brandenburg, where yields are often limited by water deficits and significant portions of crops, mainly maize, are grown for energy production. Various land use scenarios were evaluated to test the sensitivity of the methodical approach to different types of land use changes (Gutzler et al. 2014). Here, we examined the effects of increased energy cropping and increased irrigation. The economic constraints are defined as scenarios of governmental support for the different agricultural development options. Three scenarios are compared: (i) business as usual, (ii) irrigation of primary crops, (iii) and continued subsidization of bioenergy production.

## Material and methods

### Case study of Brandenburg

**Fig. 1:**
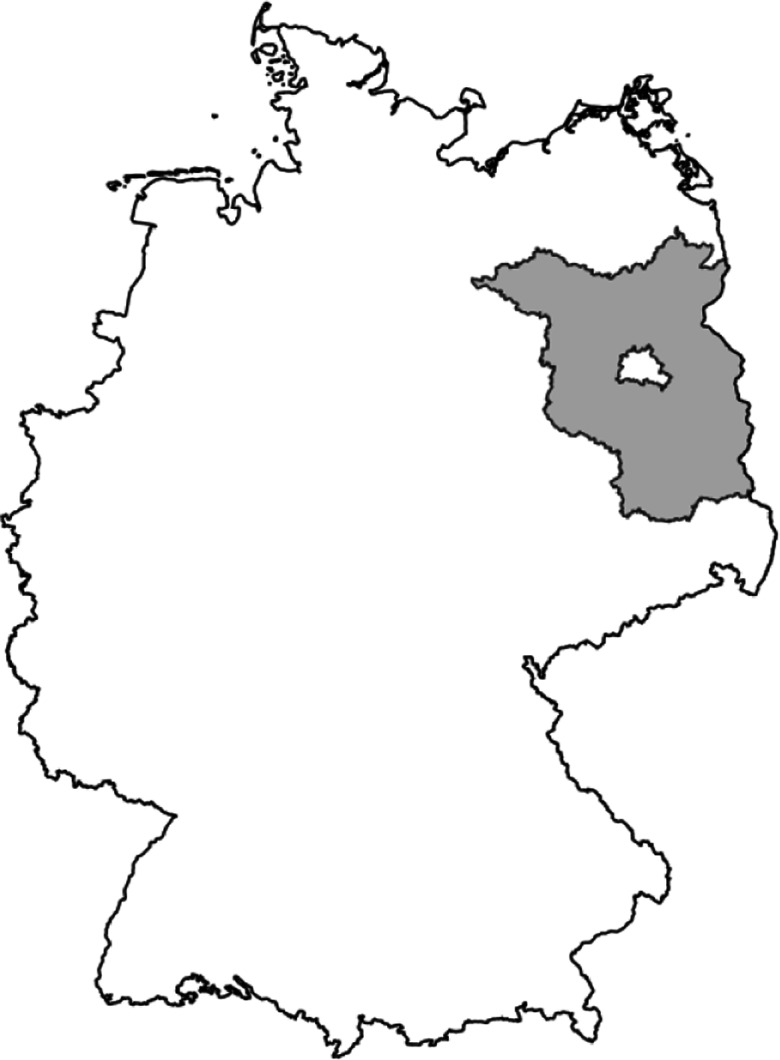
Location of the federal state of Brandenburg

Brandenburg (Fig. 1) is the fifth-largest German state with a size of 29,500 km^2^, 45 % of which is agricultural land and 37 % forest. Nearly one third of the total area is contained within conservation areas. Brandenburg hosts 620 protected areas according to the flora–fauna–habitat (FFH) directive and 27 bird reserves according to the European directive (special protected areas (SPA)) (MUGV 2009). Despite the large number of areas under protection, half of the populations of farmland birds declined by at least 20 up to 90 % between 1995 and 2008 (Langgemach and Ryslavy 2010). While typical indicators for land use intensity, such as the application of pesticides and fertilizers, remained relatively unchanged during the analyzed time period, other changes in management, such as changes in crop species composition and reductions in crop species diversity, are assumed to be relevant to the declines in farmland birds (Langgemach and Ryslavy 2010).

The landscapes in Brandenburg have been shaped by glacial and post-glacial processes. The soil conditions and landscape structure are typical for the northern portion of central continental Europe. The broad range in soil fertility and highly heterogeneous distribution determine the regional agricultural land use capabilities. The agricultural production is limited by annual precipitation (450 to 600 mm per year with frequent periods of drought, especially in early summer) and by the predominance of loamy sand or sandy loam soils with limited yield potentials. The agricultural area in the study region has been classified according to the soil yield potential and cropping options (Müller et al. 2013) into five different soil rating classes (Table 1). The four dominant crop species are winter rye, maize for silage, winter wheat, and winter rape, which together cover 64 % of the total utilized agricultural area. Irrigation is currently used only on 2 % of the cropland area because of the high costs for investment and maintenance. Since irrigation improves yields and profit margins for energy crops (Schittenhelm 2010), irrigation is expected to become more important in the case study region in the near future.

**Table 1:** Distribution of the agricultural site conditions over the study area

Soil rating class (SRC)	Share on total UAA	Soil quality number (max 100)	Dominant crops
1	7	>45	Winter wheat, sugar beets
2	22	36–45	Winter wheat, barley, winter rape, suitable for sugar beets
3	37	29–35	Winter rye, potatoes, limited suitability for winter rape, barley
4	27	23–28	Winter rye, potatoes, limited suitability for maize
5	7	<23	Winter rye, lupin

From Hanff et al. (2010)

*UAA* utilized agricultural area in %

The financial promotion of renewable energy, which started in 2004 (BMU 2004), has resulted in an intense growth of technical capacities for producing energy from biomass in agriculture. The number of biogas plants increased eightfold in Brandenburg from 2004 to 2011. Because maize is the most profitable feedstock for biogas production, the cropping area of maize increased by 50 % from 2004 to 2011, which is similar to the German average.

### Scenarios

Based on current societal and economic pressures, we chose three scenarios of future cropping practices to evaluate the sensitivity of environmental indicators to regional land use. The definition of the scenarios was based on a few assumptions for the following transregional driving forces: high world market prices for agricultural goods, an increase in problems associated with summer drought (as a predicted regional effect of climate change), and the continued fostering of renewable energy production through national subsidy programs. As a baseline for the assessments, we used a business as usual (BAU) scenario. The BAU scenario extrapolated the ongoing progress in plant breeding programs for increases in the yields of crops into the year 2025. The BAU scenario was contrasted with an irrigation scenario and an energy scenario. The detailed assumptions for all the three scenarios are summarized in Table 2. More details are provided in Gutzler et al. (2014).

**Table 2:** Assumptions describing the cropping practices for the three considered scenarios (mod. after Gutzler et al. 2014)

Parameter	Scenario			
	Reference	Business as usual	Irrigation	Bioenergy
Year	2011	2025	2025	2025
Price/cost (relative to 2011)		Net margin from agricultural goods +10 %	Net margin from other agricultural goods +10 %	Net margin from agricultural goods +10 % and net income from silage maize +20 %
Irrigation (crops)	No irrigation	No irrigation	Maize, winter wheat, winter rapeseed, sugar beet	No irrigation
Fallow land	4 %	Reduced from 4 % of cropland area in 2011 to 2 % in 2025		
Yield developments (relative to 2011; the range reflects the variation in site quality)	Statistical data	Silage maize +1.2 to +1.8 t ha^−1^		
		Winter barley +0 t ha^−1^		
		Winter rapeseed +0.55 to +0.6 t ha^−1^		
		Winter rye +0 to +0.15 t ha^−1^		
		Winter wheat +0.45 to +0.6 t ha^−1^		
		Sugar beet +7.5 t ha^−1^		
Yield increase through irrigation (relative to 2011)	None	None	Silage maize +43 %	None
			Winter rapeseed +7 %	
			Winter wheat +18 %	
			Sugar beet +18 %	
Animal husbandry (relative to 2011)	Statistical data	No change	No change	No change
Technology (relative to 2011)	Expert data	No change	No change	No change
Restrictions on crop rotations	For 50 % of winter rape, winter barley is cultivated as a precrop. The sugar beet area remains unchanged (long-term contracts)			

### Model structure

#### General approach

The initial working hypothesis is that the habitat quality of farmland is strongly influenced by land use changes, here, by changes in the composition and distribution of crops and their related management practices. The spatial distribution of crops is an economic decision that depends on land quality, available resources, preferences of the decision-making farmers, frame conditions such as the agro-environmental programs, and market conditions (access to markets, factor prices, and product prices). The spatial variation in responses to changing frame conditions may lead to varying ecological sensitivities.

The main methodological steps are the following:

1. Analysis and selection of future trends to be considered (with stakeholder involvement)
2. Translation of the trends into framework conditions for different scenarios (scenario definition)
3. Allocation of land use options under the scenario conditions (economic assessments based on a regional, linear programming farm model)
4. Assessments of land management impacts for the particular scenarios based on farmland bird indicators
  - Identification of relevant crop management activities
  - Assessment of the impacts of relevant crop management activities on farmland birds
  - Aggregation of crop management activities into one farmland bird indicator per land use option and soil rating class
  - Application to land use patterns obtained from the economic scenario analysis
5. Evaluation of the resulting field bird indices
  - Comparison with data on natural inventories (as proxies for regional sensitivities)

**Fig. 2:**
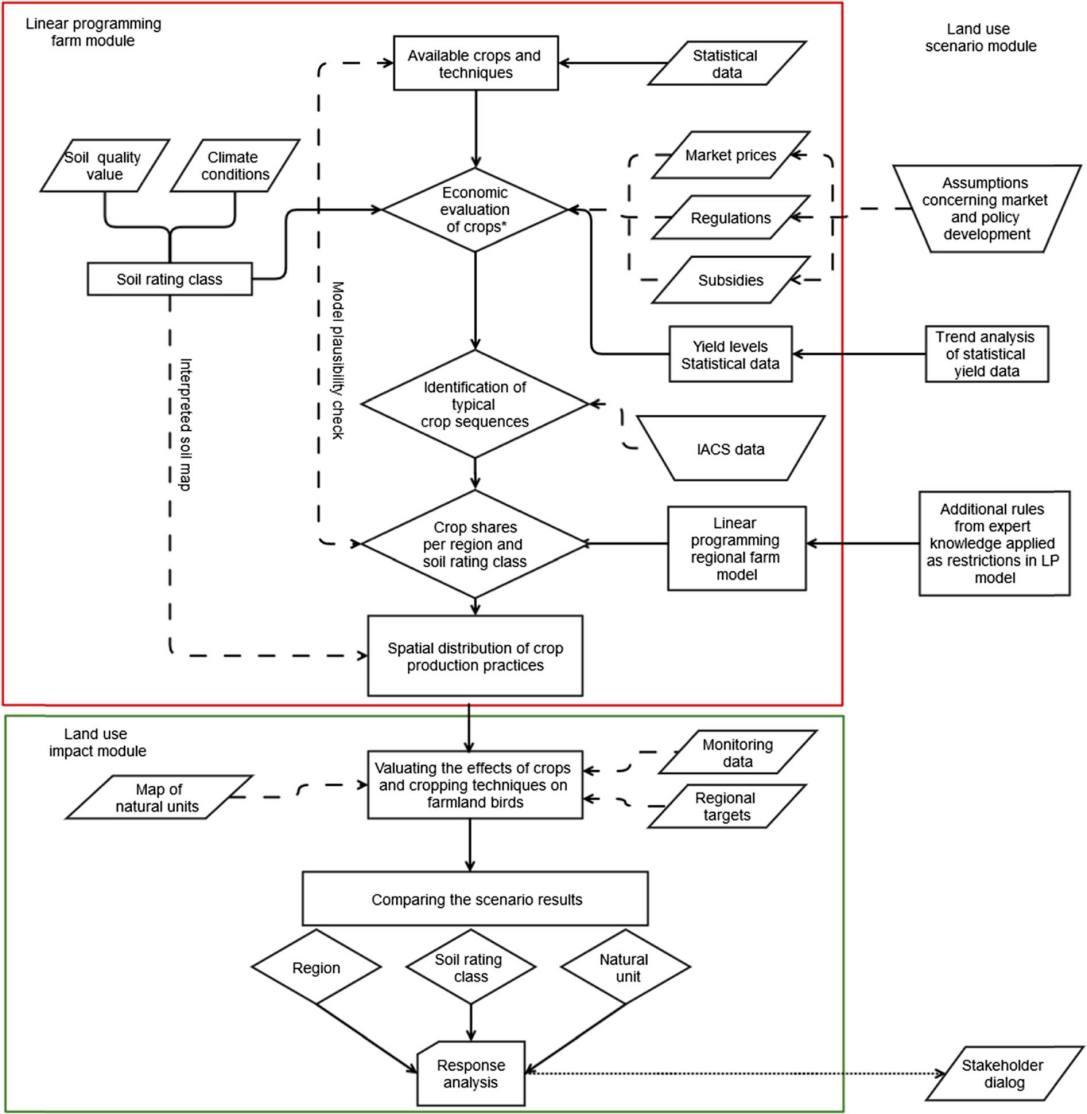
Analytical framework for the regionalization of land use impacts applied to the 14 counties in the state of Brandenburg

Figure 2 shows the links among the economic approach and the assessments of the farmland bird indices.

#### Economic assessment

The economic analysis assesses the impacts of internal and external land use drivers on cropping practices and crop distributions based on scenario assumptions and site characteristics. We used a simplified linear programming optimization model for “region farms” to represent the economic decision-making of farmers in accordance to Rounsevell et al. (2003). Each region farm represents one of the 14 Brandenburg counties and disposes of all farm resources as one county, taking into account established farm structures, including total livestock, and the amounts of area within the various soil rating classes. The approach used the existing crop production data for each of the five soil rating classes and additional expert assessments of inputs and outputs for novel cropping practices, including irrigation. The net margins of crops were compared based on default machinery cost figures (KTBL 2012). A linear programming farm model was constructed for each county using MS Excel^©^. The assumptions and constraints were (i) constant livestock fodder requirements, (ii) complete use of manure in the cropping systems, (iii) constant levels of contract-based cropping systems, and (iv) adherence to crop rotation restrictions. On this basis, the model maximized the total net margin by district by allocating all portions of the area of every soil category to the best-performing cropping practices when taking the restrictions into account.

The result was a specific distribution of crops for each county and for each of five soil categories. Crop yields were calculated by using the statistical hybrid model *YIELD* estimation based on *STAT*istics (YIELDSTAT) (Mirschel et al. 2014). The matrix contained 16 agricultural crops and two grassland types. A crop species-specific correction algorithm was used to calculate the yield effects of irrigation, of crop rotation, and of different soil tillage methods, such as conventional tillage. Moreover, the overlaying trends for the progress in agro-management and plant breeding observed to date and predicted to 2025, as influenced by genetic progress, climate change, and management improvements, were taken into account. The data only covers conventional farming systems.

#### Assessment of land use impacts on farmland birds

We chose the “indicator for species diversity and landscape quality” as used within the German National Biodiversity Strategy (BMU 2007). The indicator aggregates the population trends of ten bird species typical for agricultural land, hereinafter to be referred to as “farmland bird indicator.” Three of these species (Red kite (*Milvus milvus*), Little owl (*Athene noctua*), Black tailed godwit (*Limosa limosa*)) are related to specific forest structures or to grasslands and were excluded. We distinguished two groups of birds: (i) birds that breed on arable land (skylark (*Alauda arvensis*), corn bunting (*Miliaria calandra*), lapwing (*Vanellus vanellus*), and winchat (*Saxicola rubetra*)) and (ii) birds that only feed on arable land (red-backed shrike (*Lanius collurio*), the yellowhammer (*Emberiza citrinella*), and the woodlark (*Lullula arborea*)).

**Fig. 3:**
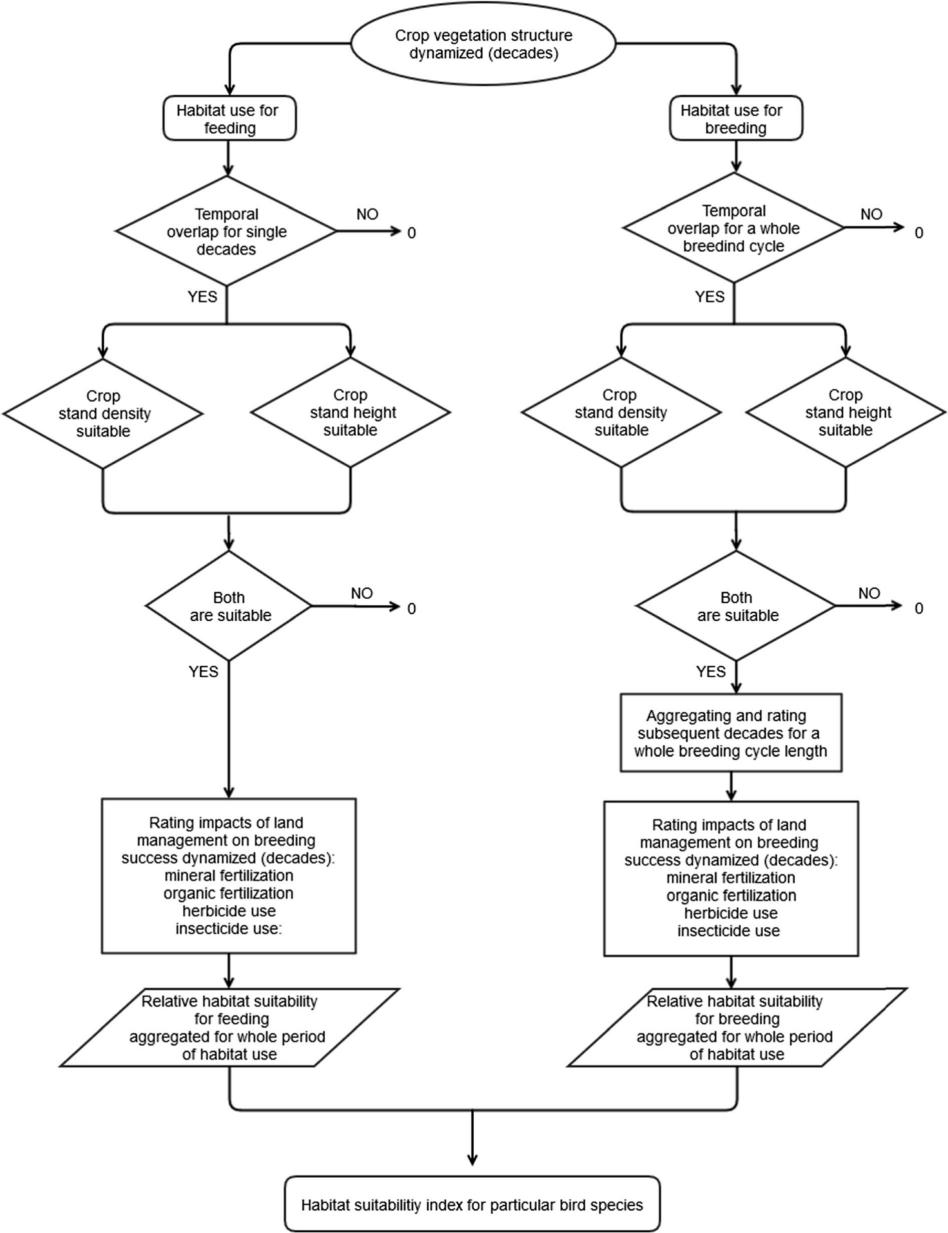
Detailed description of the land use impact module used to determine the habitat suitability of crops for farmland birds

The land use impact module addresses the changes in crop species and crop management on the habitat availability or suitability for wildlife species that use arable farmland as their main habitat. This module is based on the basic assumption that crops serve as “foundation species” (according to Ellison et al. 2005) for the farmland birds by predefining time spans for using crops as habitat and the habitat quality (microclimate, shadowing, accessibility) for wildlife species. Anderson and Fergusson (2006) have described this as the “intrinsic biodiversity value of the crop itself.” The habitat value for each bird species is calculated separately for combinations of crops cultivated using their typical regional production technique. Then, the values for the single species are aggregated to mean values and scaled to 1 for the breeding and feeding birds. The habitat index value of 1.0 is the maximum reachable value, indicating high habitat suitability for the specific group of bird species. The habitat quality of a certain crop is assessed by contrasting the habitat characteristics provided by the crop with the habitat demands of the bird species (Tables 3 and 4; Fig. 3). The calculation of the habitat suitability begins with the selection of a species and the definition of their habitat demands. The assessment consists of three subsequent steps that calculate the following: (1) the match between the crop growing period of the crop and the reproduction period for the target species, (2) the temporal match between the demands for appropriate vegetation structure by the target species and the provision of such structure by the crops, and (3) the effects of disturbances by the crop management techniques implemented during the habitat usage of the target species. The methodology is described in further detail by Brandt and Glemnitz (2014).

**Table 3:** Characteristics of crops and crop management activities and related requirements/sensitivities of farmland bird species as the basis for assessing the habitat suitability of crops fields

Characteristic of the crop and crop management	Requirements/sensitivities of the bird species
Time and length of growing period	Number, time, and length of breeds
Time and length of growing period	Feeding time and length
Vegetation structure (vegetation height and density)	Habitat preferences (vegetation density and height)
Dates of disturbances by farming activities (sowing, soil tillage, fertilization, and pesticide usage)	Susceptibility to type and time of disturbance

**Table 4:** Preferred crop stand properties for the breeding and feeding activities of farmland bird species

Species	Breeding period	Feeding period
Skylark	Density^a^ (35–80 %); height (20–50 cm)	Density^a^ (35–80 %); height (20–50 cm)
Corn bunting	Density^a^ (70–100 %); height (50–120 cm)	Density^a^ (50–100 %); height (30–120 cm)
Lapwing	Density^a^ (0–50 %); height (0–10 cm)	Not relevant
Winchat	Density^a^ (30–60 %); height (0–30 cm)	Density^a^ (30–60 %); height (0–30 cm)
Red-backed shrike	Does not breed on fields	Density^a^ (0–50 %); height (0–70 cm)
Yellowhammer	Does not breed on fields	Density^a^ (50–90 %); height (0–70 cm)
Woodlark	Does not breed on fields	Density^a^ (25–50 %); height (5–50 cm)

Species selection refers to the farmland bird index from the German biodiversity strategy. Data source: Fuchs and Matthews (2008)

^a^Measured as vegetation coverage

Crop fields with tall vegetation layers may act as sink habitats for farmland bird populations when habitat conditions change during the breeding period (Arlt et al. 2008; Chamberlain and Fuller 2000). Due to the lack of quantitative data on this effect in the literature, we developed a scoring scheme for the temporal dynamics of the habitat conditions during the breeding season (Table 5, upper section). In the absence of direct relationships among pesticide use, breeding success, and food availability for farmland bird species, a scoring scheme was also used to relativize the structural habitat quality for feeding birds. This scoring scheme (Table 5, lower section) follows the logic that various farming practices (e.g., pesticide use) have “cascading” effects on birds (Hallmann et al. 2014; Boatman et al. 2003). Fertilization is not regarded to have any direct impact on farmland bird populations, except for the relatively new practice of fertilizing with biogas slurry. The application of biogas slurry on the soil surface is a new technique that is typical of energy cropping techniques and may destroy bird nests as well as cover weeds and arthropods. The temporal dynamics of the habitat suitability were assessed in periods of 10 days (“decades”). For the calculation, a number of regionally typical production techniques were as used in the economic farm model considered for each crop, taking into account various subsequent crops and the regional crop species composition. The definition and the timing of the crop production techniques were gained from local experts. Due to the interdependencies between crop species and the production techniques, the evaluation followed a hierarchical structure in which the production techniques were even assigned to particular crop/precrop pairs and the crop pairs were allocated to the soil rating classes. For the completion of a brood, it is essential to have suitable habitat conditions for the entire breeding period; otherwise, the crops might serve as a sink for the population. We assumed a certain adaptability of the species to remain at a plot when the habitat conditions switched to unfavorable conditions at the end of the breeding periods by giving a reduced score, depending on the length of suitable vegetation structure (Table 5, upper section). The number of potentially successful broods was averaged for the four breeding species whereas the number of decades accessible for feeding was averaged for the three foraging bird species. These values were calculated for each crop production technique (*N* = 126) and every bird species (*N* = 7) and then transferred to the soil rating classes by calculating area-weighted averages for the crop composition of the farm units of a particular region. Species behaviors and habitat preferences were parameterized (Table 4) using a comprehensive literature review (Fuchs and Matthews 2008) focused on northeast Germany. The phenological data for the crop stand (height and coverage) input data were obtained from comparative vegetation surveys for 14 crops that were conducted at the Güterfelde experimental station (near Berlin) from the years 2005 to 2012.

**Table 5:** Scoring scheme for the habitat suitability of various crop species for bird species, depending on the function of arable land for nesting and breeding or for feeding

Nesting and breeding		Feeding	
Length of habitat suitability of the crop vegetation			
Three decades	10	Each decade	10
Two decades	7		
One decade	3		
Disturbances			
Mineral fertilization	±0	Mineral fertilization	±0
Organic fertilization (biogas slurry)	−5	Organic fertilization (biogas slurry)	−5
		Herbicide use	−5
		Insecticide use	−10

See Fig. 3; “decade” equals ten consecutive days

### Evaluation of the results

Since many of the regional data on environmental sensitivities or on regional planning in Germany refer to a geomorphological classification scheme for Germany (Meynen and Schmidhüsen 1962), we intersected the results for the farmland bird indices with the map of geomorphological units for Brandenburg. The classification is based on geomorphology, but it integrates also information on climate and habitat inventory. The results of the scenarios have been mirrored with the latest results of the regional bird monitoring survey conducted from the years 2005 to 2009 and with the “landscape program” for Brandenburg (MLUR 2000) which specifies environmental goals, e.g., for biodiversity. The bird monitoring data were obtained from the German breeding bird atlas project (ADEBAR) for the Brandenburg area (ABBO 2011). The atlas analyzes the frequency of over 195 bird species (not only farmland birds), their population sizes, and their trends on a grid map layer (125 km^2^), which is the reference layer since the beginning of the bird monitoring.

## Results

### Farmland bird index by crop

**Fig. 4:**
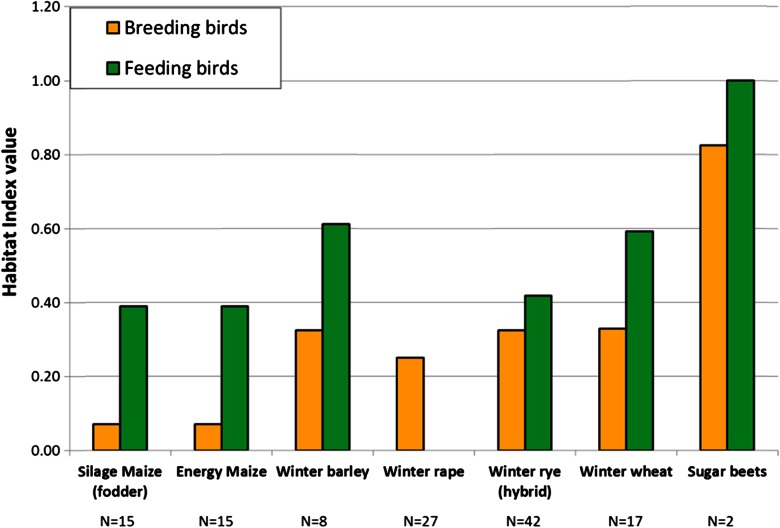
Intrinsic habitat values of particular crops for breeding and feeding farmland birds, calculated and averaged over different numbers of crop production techniques that are typical for the crops in Brandenburg (*N* number of different crop production techniques considered)

Using the land use impact module (Fig. 3), the bird species responses were calculated as a function of the crop species, its vegetation structure, and a variety of crop-related production techniques. Figure 4 summarizes these crop effects on both groups of farmland bird species. The lowest habitat quality for breeding birds was provided by silage maize and energy maize. The winter cereals (barley, rye, wheat) differ only very slightly, while sugar beets have the best structural habitat quality for both groups of birds. For the species which only feed on arable land, the differences among the crops are similar to those of the breeding birds, except winter rape, which crop stands are already too dense early in the summer and can probably not be accessed by the birds. Maize serves substantially better as a habitat for feeding birds than for breeding birds.

### Land use change

**Fig. 5:**
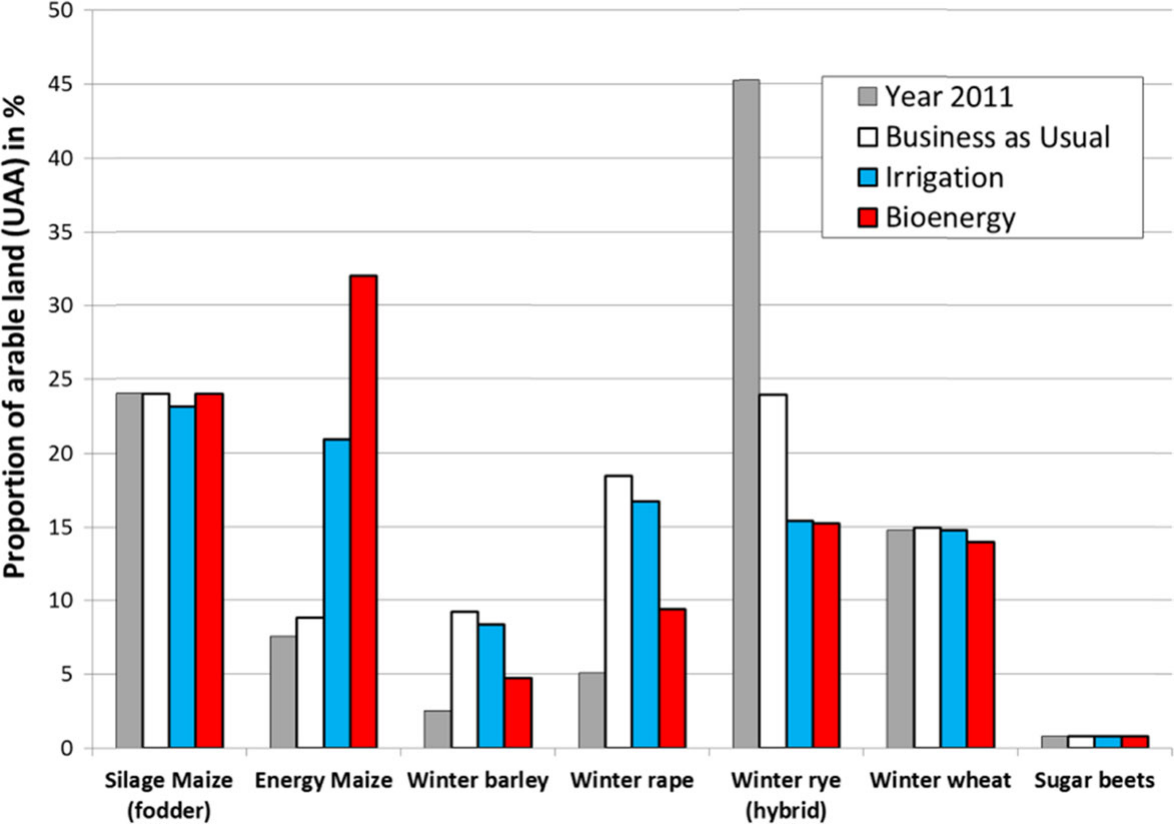
Effects of the applied land use scenarios on the proportions of the dominant crops for the whole case study area, as compared to status quo in 2011

The applied land use scenarios (Table 2) modify the relative economic attractiveness among the crops and thus lead to changes in the crop composition within a region (Fig. 5). Even the continuation of the farming under the current economic frame conditions to the year 2025 (“BAU” scenario) resulted in a serious increase in the cropping area for rapeseed and winter barley at the expense of winter rye compared to the status quo in 2011. The propagation of irrigation increases the profitability of maize cropping and further reduces the winter rye area. The energy cropping scenario results in a tripling of the maize cropping area. The cropping area of winter barley and winter rape thus decreases in this scenario.

**Fig. 6:**
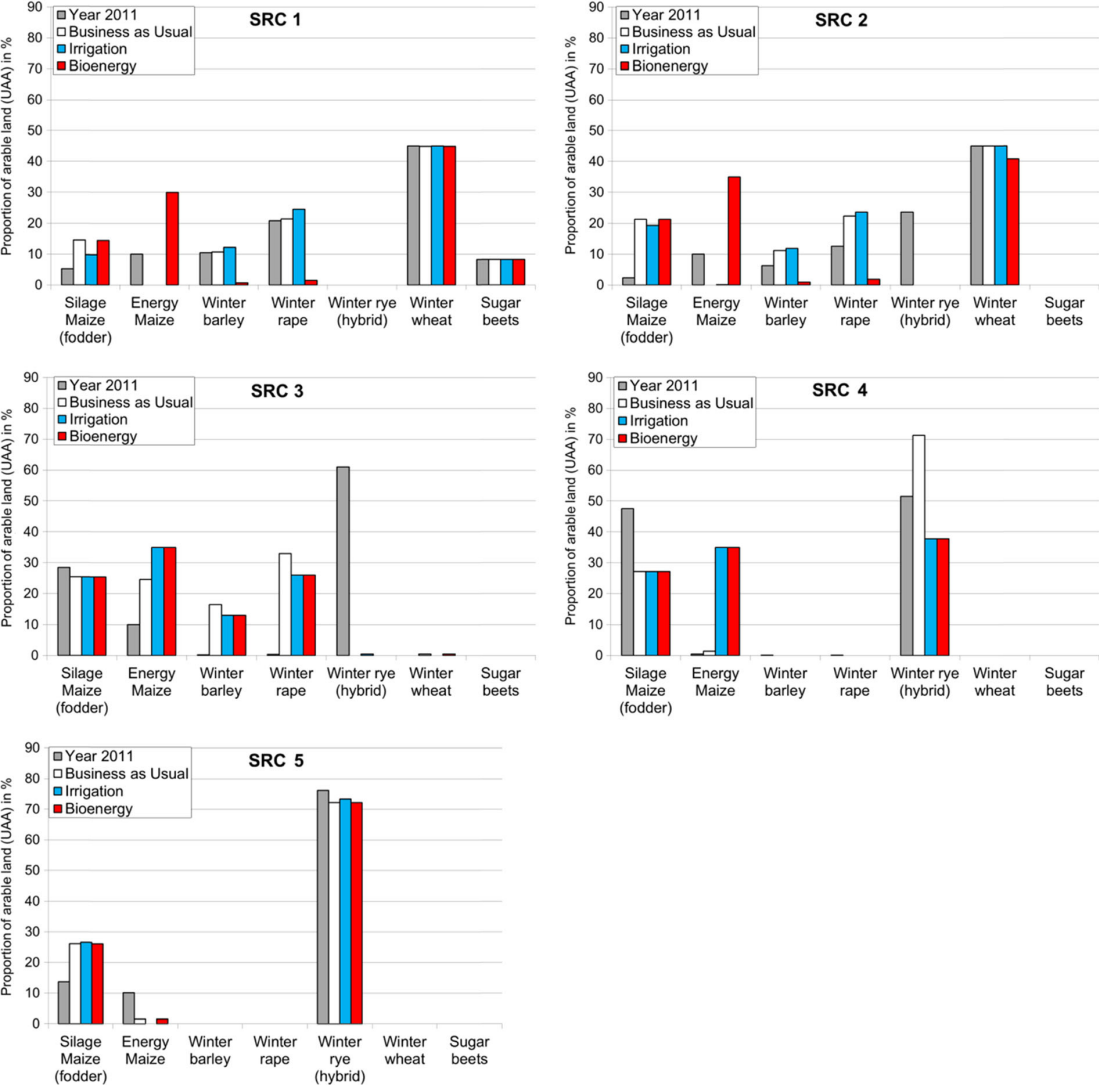
**a**–**e** Relative proportions of the cropping area of the predominant crops for the considered land use scenarios, contrasted with the status quo in 2011 (output of the economic module, graphed by soil rating class (SRC), from most fertile, SRC1, to least fertile, SRC5)

For all three scenarios, there are significant crop yield differences among the soil rating classes, which result in divergent solutions for the net gross optimization by the regional farm module. The resulting crop distribution patterns as aggregated for the different soil rating classes are shown in Fig. 6a–e. Interestingly, the extent of changes in the proportion of crops is related to the soil quality in a “bell-shaped manner: Low impact in the least and most fertile areas, significant changes were found in between. This applies for all scenarios. The scenario assumptions cause only slight changes in the areas of the main crops for the most fertile regions (SRC1); in particular, the changes caused by the irrigation scenario are minimal. Economic support for bioenergy increases the maize cropping area, which replaces winter barley and winter rape in SRC1. In the SRC2 and SRC3, winter rye disappears completely even when continuing BAU. In SRC2, irrigation slightly increases the cropping area of winter barley and winter rape, but not the area dedicated to energy maize. Energy maize benefits strongly from the bioenergy scenario. The increase in energy maize reduces the areas of winter barley and winter rape to nearly zero and even slightly decreases the winter wheat cropping area. Irrigation increases the profitability of energy maize cropping within the SRC3 and SRC4, even without additional subsidies. The irrigation and bioenergy scenarios have the same effects within SRC3 and SRC4. In SRC3, both scenarios reduce winter barley and winter rape cropping slightly. In SRC4, energy maize cropping increases at the expense of winter rye cropping. In regions with very low soil fertility (SRC5), neither irrigation nor financial support for bioenergy is sufficient to change the profitability among the crops; winter rye remains the most stable in terms of yield within these regions.

### Bird species response

**Fig. 7:**
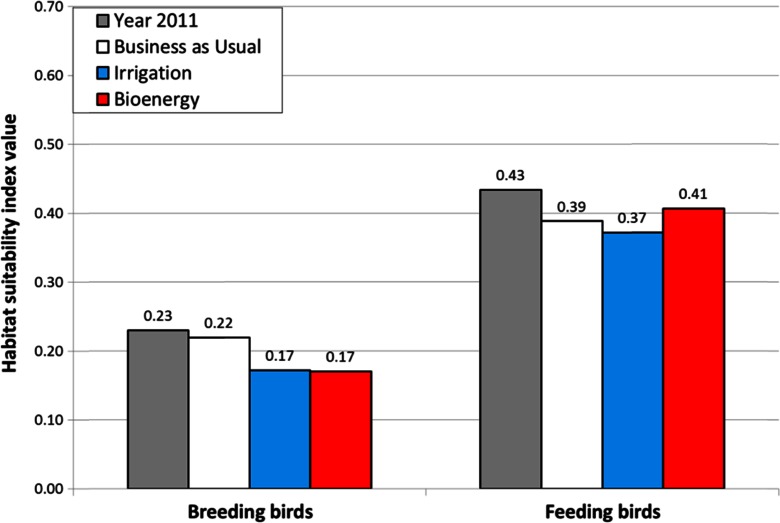
Effects of land use scenarios on the mean habitat suitability index for farmland birds in Brandenburg (area-weighted averages calculated for the cropping area of particular crops and their related cropping techniques)

The economic farm model allocated the crops with the most efficient crop production techniques to the soil rating classes of the counties in the case study region. By valuating the changes in crop species composition using the crop-related habitat suitability index, we examined how the land use scenarios affect the habitat availability for farmland birds for the separate SRCs. The overall impacts of the scenarios for the whole area of Brandenburg are shown in Fig. 7. For breeding birds, we found only a slight deterioration with the BAU scenario. In comparison, the increased irrigation and support for bioenergy have strong negative effects on the habitat suitability for farmland birds. The effects for the feeding birds are less dramatic. For feeding birds, the propagation of maize cropping produced the same-sized effect as the BAU scenario. Most of the decline in the habitat suitability for feeding birds appeared to be related to the decline in the rye cropping area. The irrigation scenario resulted in a lower overall habitat quality for feeding birds.

### Regional variation in bird species indices

#### Differences among soil rating classes

The variation in the scenario impacts on the cropping structure among the soil rating classes (see Fig. 6a–e) necessarily indicate diverging results for the habitat suitability for the farmland birds. Table 6 summarizes the mean changes for the particular soil rating classes. The evaluation was based on the habitat suitability indices for the single crops and their transfer to the regions within the area of the case study. Arable land of SRC3 and SRC4 exhibited the strongest declines overall. While the habitat suitability for birds declined by between −15 and −10 % on half of the area of SRC2, the main share (73.3 %) of the area of SRC4 exhibited declines of −45 % and greater (Table 7).

**Table 6:** Area-weighted breeding bird indices for the “business as usual” scenario and the relative changes (in %) for the irrigation and bioenergy scenarios for Brandenburg (output of the land use impact module)

Soil rating class	Business as usual	Change in %	
		Irrigation	Bioenergy
SRC1	0.33	−8.80	−9.08
SRC2	0.28	−11.54	−13.42
SRC3	0.14	−20.75	−20.77
SRC4	0.23	−47.02	−47.02
SRC5	0.23	1.53	−0.01

**Table 7:** Transition matrix for the relative changes in the indices for breeding birds for the “irrigation” and “bioenergy” scenarios compared to the “business as usual” scenario for Brandenburg (% of total area for each SRC)

Index change (%)	“Irrigation” scenario					“Bioenergy” scenario				
	SRC1	SRC2	SRC3	SRC4	SRC5	SRC1	SRC2	SRC3	SRC4	SRC5
≥0	–	–	19.0	–	98.5	2.6	–	19.7	–	98.5
−0.1 to −15.0	100	100	5.0	–	1.5	97.4	81.0	5.1	–	1.5
−15.1 to −30.0	–	–	49.6	5.2	–	–	19.0	49.6	5.2	–
−30.1 to −45.0	–	–	25.5	21.5	–	–	–	25.5	21.5	–
<−45.0	–	–	–	73.3	–	–	–	–	73.3	–

**Fig. 8:**
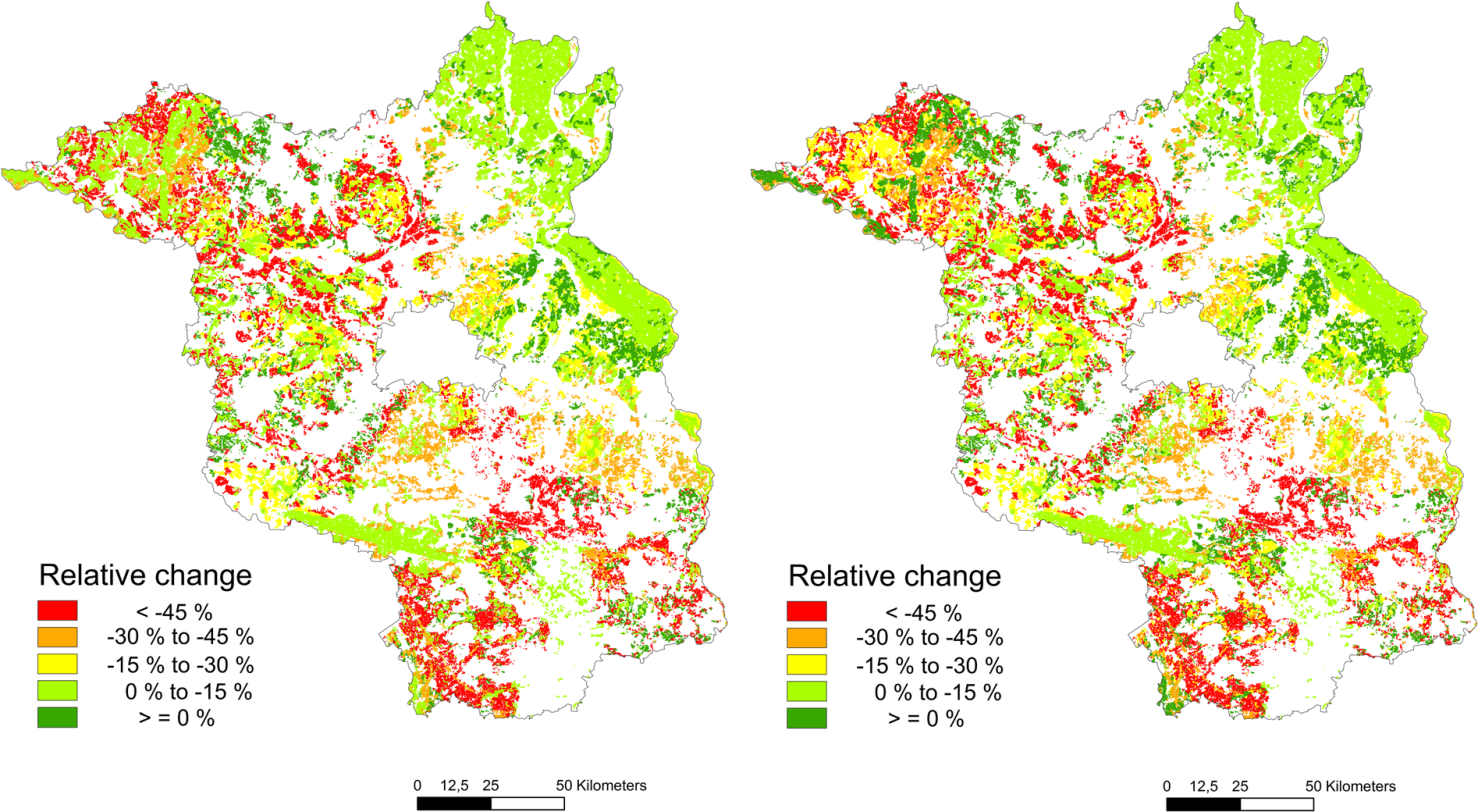
Regional distribution of the impacts of the “irrigation” (*left*) and “bioenergy” (*right*) scenarios on the index for breeding birds across Brandenburg compared to the “business as usual” (=100 %) scenario (map basis: distribution of soil rating classes)

Since the distribution of the different soil rating classes is highly variable within Brandenburg, the regional impacts are also unevenly distributed. The regional distributions of the changes in the breeding bird indices for the “irrigation” and “bioenergy” scenarios compared to the BAU scenario are shown in Fig. 8. The maps reveal large regions with a low or moderate decline in the index for breeding birds, e.g., for the northeast of Brandenburg, while other regions exhibit a dramatic (−45 % and more) decline in the habitat quality for breeding birds.

#### Comparisons with regional bird inventories and regional environmental planning goals

Comparing the scenario results with the monitoring data and planning targets provides additional information for the interpretation of the indicated changes. The most recent bird inventory for Brandenburg (ABBO 2011, Fig. 9a) notes that the overall species diversity varies regionally between 100 and 151 species. Hot spots for bird species diversity can be found in the northeast, the midwest, and the southeast of Brandenburg. The seven farmland bird species addressed in our study are classified as “common” or “very common,” abundant on 96–100 % of the monitored area. Only the lapwing exhibits stronger regional limitation, occurring in only 83 % of the monitored grid cells. The mean breeding pair density and its regional distribution vary among the species. While the skylark, the red-backed shrike, the yellowhammer, and the winchat are more or less evenly distributed spatially over the regions, the corn bunting has the highest densities of breeding pairs in the eastern part of the area, the winchat in the middle and northeast of Brandenburg, and the woodlark in the south.

**Fig. 9:**
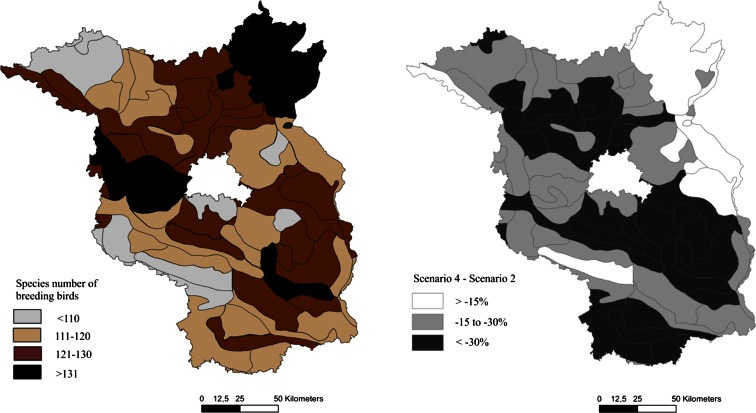
**a**, **b** Comparison of the patterns of the bird species numbers over Brandenburg from the results of the bird monitoring survey from 2005 to 2008 (modified from ABBO 2011, *left*) with the calculated relative change in the index for breeding birds for the “bioenergy” scenario (“business as usual” scenario = 100 %, for the geomorphological units after Meynen and Schmidhüsen 1962)

**Fig. 10:**
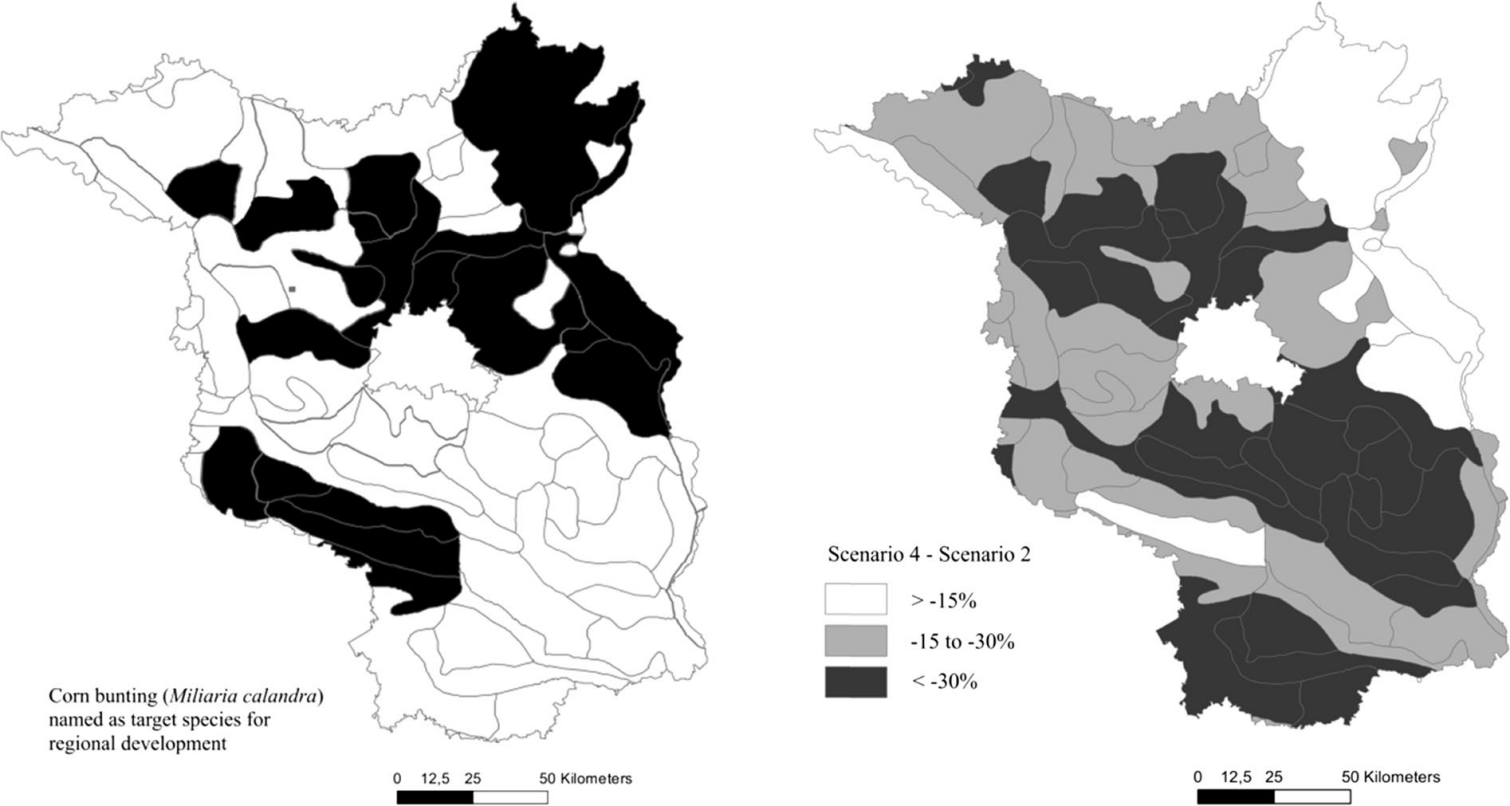
**a**, **b** Comparison of the regions where the corn bunting was named as a target for regional species conservation (landscape program for Brandenburg, MLUR 2000, *left*) and the relative change in the index for breeding birds for the “bioenergy” scenario (“business as usual” scenario = 100 %, for the geomorphological units after Meynen and Schmidhüsen 1962)

Of the set of bird species included in the farmland bird indicator for Germany, only the winchat and the lapwing are classified as strongly endangered (ABBO 2011). No species protection program has been adopted for either farmland birds in general or for any single farmland bird species in Brandenburg. The landscape program for Brandenburg (MLUR 2000) details the conservation objectives and the guidelines for sustainable development and serves as a contextual framework and directive for all planning activities conducted at regional or local scales. Within the landscape program of Brandenburg, the corn bunting is named as a target species for nature conservation in a number of regions, most likely as a proxy for other farmland birds. According to this document, the corn bunting is particularly worthy of protection, especially in the northeastern and central parts of Brandenburg (Fig. 10a).

The projection of the scenario results to the geomorphological units of Brandenburg (Figs. 9b and 10b) allows for a comparison with the regional bird inventory and the landscape program (Figs. 9a and 10a). The differences between the irrigation and bioenergy scenarios are slight in terms of absolute value and regional distribution. The decline in suitable habitats within arable land for farmland birds will be strongest in the central and southwestern parts of Brandenburg, partially in regions where the species richness of birds is classified as medium high or low (Fig. 9a, b). Conflicts between conservation needs or intentions for farmland birds can be identified in central Brandenburg, where the habitat suitability declines considerably but improvements in the habitat conditions for the corn bunting, for example, have been declared as an objective for regional development (Fig. 10a, b). In most of the regions with strong declines in habitat suitability, farmland birds are not the focus of the landscape program for Brandenburg. The changes in agricultural management in the northeast or east might only have slight effects on the breeding birds, but stronger negative effects for the feeding birds.

## Discussion

The presented habitat indices have been developed to amend the assessment of the impacts of changes in cropping patterns on farmland birds. These indices address measures that can be influenced by the farmers directly: the crop choice and crop production technique. The ways that these drivers and structures are implemented determine how realistic the response of the model will be to the considered scenario. Farmland birds respond to changes in numerous factors, such as landscape structure and crop management actions (Concepcion and Diaz 2011; Firbank et al. 2008). One limitation of our specific focus is the disregard of scale effects. Marja et al. (2013) found the variation explained between farmland bird variables and landscape metrics to increase with the size of the study area. Similarly, Böhning-Gaese (1997) revealed that the impact of different habitat types in predicting bird species richness changed with increasing raster sizes (grain) for spatial analyses. Especially, the habitat occupancy of habitat specialists and rare species seems to be better interpretable at larger scales, e.g., the landscape scale (Skorka et al. 2006). Farmland birds need larger areas than the nesting area for survival and successful reproduction. The size of these activity territories varies not only between different species. Even for a certain species, the size of territories is influenced by food availability, predator densities (Tryjanowski et al. 2002), interspecific and intraspecific competition, and other factors. For skylarks, Hiron et al. (2012) reported regional differences in temporal habitat shifts among crops, which might not only be influenced by the availability of alternative habitats in the surrounding but also by the availability of different crops. Comparing the evidence from the various effects observed at multiple scales, landscape structure explained most of the variation in farmland bird abundances in a study by Siriwardena et al. (2012). Field boundaries and margin habitats were highly correlated with the number of breeding species and total community density in the studies of Tryjanowski (1999) and Siriwardena et al. (2012). In a study across Europe (Guerrero et al. 2012), factors such as the diversity of land use categories, crop diversity, and field sizes accounted for most of the variation in ground-nesting farmland bird individual and breeding pair densities, while factors related to crop management, such as the number of pesticide applications and yield levels, had a significant influence only on the density of skylark breeding pairs. The diversity of crops and cropping practices remains important from a management perspective because they are highly volatile and drive the population densities of farmland birds (Siriwardena et al. 2012; Langgemach and Ryslavy 2010). However, hierarchical approaches considering different spatial scales (e.g., field–landscape–region) as well as their interactions might be useful to address scale impacts (see, e.g., Saab 1999).

There is growing evidence that the environmental effects of cropping interact with the surrounding landscape (Tscharntke et al. 2005; Dauber et al. 2010; Gevers et al. 2011). Farmland birds have large home ranges and are, at least temporarily, able to switch to adjacent areas for feeding in cases of disturbance (Chamberlain et al. 2000). The intraseasonal switches in habitat use seem to be widespread among farmland birds and hence might have critical implications for their conservation (Brambilla et al. 2012). Since the relevance of different landscape structures varies considerably among species (Brandt and Glemnitz 2014), the combination of cropping effects with the effect of landscape structures is logical, first of all, for modeling the response of single species. Even in that case, a modular model structure would promote the visibility of the causal effects of the different factors. Agent-based models provide a precise outlook for population trends and interactions with the landscape context (Casado et al. 2014; Gevers et al. 2011) but, to date, are limited to sets of single species and single landscapes or catchments.

Our results demonstrate the sensitivity of the developed habitat indices for evaluating the impacts of land use changes at the level of crop selection or production techniques for farmland birds. The trend and effect size of our findings are in line with those of Smethurst et al. (2005) and Ladner et al. (2007). ABBO (2011) expects the recent changes in cropping areas among crops and in cropping techniques to drive reductions in the skylark populations down to 50 % in Brandenburg in the next years. Little is known concerning the abilities of various farmland birds to adapt to changing habitat conditions or disturbances. Negative impacts of pesticides on population of farmland birds could be only verified for the partridge (Boatman et al. 2003). For the yellowhammer, there was only a probability of brood reduction reported. Even the negative correlations between neonicotinoids and birds (Hallmann et al. 2014) are difficult to explain (Goulson 2014) or to quantify in terms of a cause–effect relationship. Thus, progress in quantifying the effects of cropping techniques on population sizes or brood success could improve significantly the employed rating scales and, thus, the quality of assessments. To date, it has been difficult to prove such relationships even with manipulative experiments (Goulson 2014).

Taking into account the qualitative changes within the land cover types is a necessary and challenging task to improve biodiversity and ecosystem service assessments (Haines-Young 2009). There are plenty of results available from field research demonstrating the importance of the choice of agricultural production techniques for determining environmental effects on biodiversity. One of the reasons why this knowledge has not been well integrated into regional assessments is the issue of scale effects (Legendre and Legendre 1998). The scales addressed directly by the production techniques are the plot and the single year. However, regional assessments act on larger scales and require data availability for the entire area and time periods of several years. The presented methodology attempts to bridge these scales in order to further develop impact assessment methodology and to contribute to regional land use assessments. The methodological innovations of the presented approach are (i) the integration of current information with a link to statistical or monitoring data, (ii) the coupling of this information to economic models to determine the effects of the spatial distribution of natural land use potentials (economic land use options or alternatives), which is also suitable for anticipating farmers’ decision-making, and (iii) the use of soil rating classes as a link between the plot-related land management and environmental effects and regional data.

A soil rating classification was used to account for any considerable differences in the physical characteristics of the landscape, such as soil type and climate, that influence land use decisions and to allow for the extrapolation of farm-scale models to wider geographical regions (Rounsevell et al. 2003). The classification applied in the present study uses the yield potential as a target indicator (Müller et al. 2013). The classification includes information on soil texture, relief, and climate in addition to soil structure and explains greater than 70 % of the variability in crop yields for a given land use system (Haines-Young and Potschin 2004). The soil quality indices can be attributed with regard to their potential for hosting various crops in regard to either surviving natural hazards (such as drought, freezing, or wetness) or returning reasonable yield levels. Because of the focus on yield levels, the resulting soil quality classes can be evaluated economically and be used in decision support systems (Zander et al. 2008) or scenario analyses. In the context of the present study, the soil quality classes provide the spatial structure for the spatial allocation of the crops and their yield potential and thus integrate the yearly variation in cropping at the plot scale. In combination with the data from official statistics, the soil rating classes can be used to identify typical crop rotations or environmental risks that may arise from agricultural cropping (Bethwell et al. 2012). Moreover, when the soil quality classes are combined with additional statistical data, e.g., the farm type, important information can be gathered regarding the land use history and the flexibility of farmers to change their land use. Garnett (2011) have demonstrated that various farm types adapt differently to climate change. Thus, a consideration of farm characteristics may contribute to a further improvement of impact assessment models. The coupling of the environmental impacts with the economic module in the presented approach was used not only to anticipate farmers’ decision-making but also to determine the indirect land use changes by identifying and quantifying the crops and crop production techniques that will be replaced by the increases in other crops. An increasing number of papers have noted the indirect effects of land use changes, especially in regard to energy cropping (Gevers et al. 2011; Dauber et al. 2010).

The economic farm model that we used to generate scenario-specific cropping patterns is a partial supply model implemented at the regional level and based on crop production activities and a linear programming approach. As the farmland bird index relates only to arable farmland, we used a largely simplified approach, considering livestock and grassland as invariant. This approach has a number of limitations related to the way existing farms, market conditions, and policies is represented in our approach and limits the applicability to scenarios with limited policy and market changes. The second set of limitations relates to the level of detail with which crop production intensity and spatial peculiarities could be included in the approach, which will impact the accuracy of the farmland bird indices. However, as the farmland bird index depends in the first place on the crop choice, which again depends largely on the type of the farm and the quality of the farmland, a more detailed representation of the farms and their available land would most likely not improve the indicator. If larger policy changes are expected or new technologies are involved, better responses to policies could be achieved by adding more detail (e.g., activities and restrictions concerning livestock and grassland) to the modeling approach (Janssen and van Ittersum 2007; Zander et al 2008).

The results of the land use scenarios clearly reveal the uneven distribution of the cropping options and cropping alternatives across the study area. In relation to the agricultural and economic options, the farmers act divergently to changes in external economic conditions, depending on the respective soil quality class of their land. The results of the economic farm module indicate that the set of available cropping options increases together with the yield potential. The less fertile arable regions are less resilient against land use changes. Bioenergy cropping has often been suggested as an option to increase crop diversity, even on marginal land. Gehrels et al. (2012) have reported that cropping for bioenergy on marginal land provides no reliable economic return and that the economic cost of the entire process largely exceeds the value of the substituted fossil fuel. In any case, cropping seems to be more vulnerable to homogenization with decreasing soil fertility. Our findings contradict the widespread notion that the environmental impacts of land management are directly negatively correlated with agricultural fertility (Guerrero et al. 2012). This relationship appears to be restricted to the plot scale, at which the cropping inputs (fertilization, plant protection) are correlated with the yield level. At larger scales, the effects of the crop choice, which is related to soil fertility gradients, may mask the effects of local-scale inputs. Crop diversity has been reported as one of the main drivers for species diversity of wildlife in agricultural areas (Smith and Gross 2007; Karp et al. 2012). In support for the promotion of biodiversity on arable lands, we have demonstrated the high importance of crop diversity in agricultural landscapes as well as the role of its spatial distribution. We found that the simplification of cropping may concentrate to agriculturally marginal regions, which are, from a structural point of view, regarded as highly valuable for biodiversity. Farmland species (e.g., birds) are not the primary focus for conservation efforts in these regions, at least in our study area.

Further research is required to quantify the amount and type of ecological compensation areas which will be needed outside of the agricultural fields to compensate for negative impacts of changes in crop management. We suggest that this will vary depending on landscape contexts.
